# Supplementary material for: Non-invasive temporal interference stimulation of the hippocampus suppresses epileptic biomarkers in patients with Epilepsy: biophysical differences between kilohertz and amplitude modulated stimulation
Source: Brain Stimul. 2026 Jan-Feb;19(1):None. doi: 10.1016/j.brs.2025.11.008 (PMC12858428; doi:10.1016/j.brs.2025.11.008)
Supplement: Multimedia component 1 [file mmc1.docx]

**Supplementary Figures:**

**Figure S1. Center-by-center analysis.**
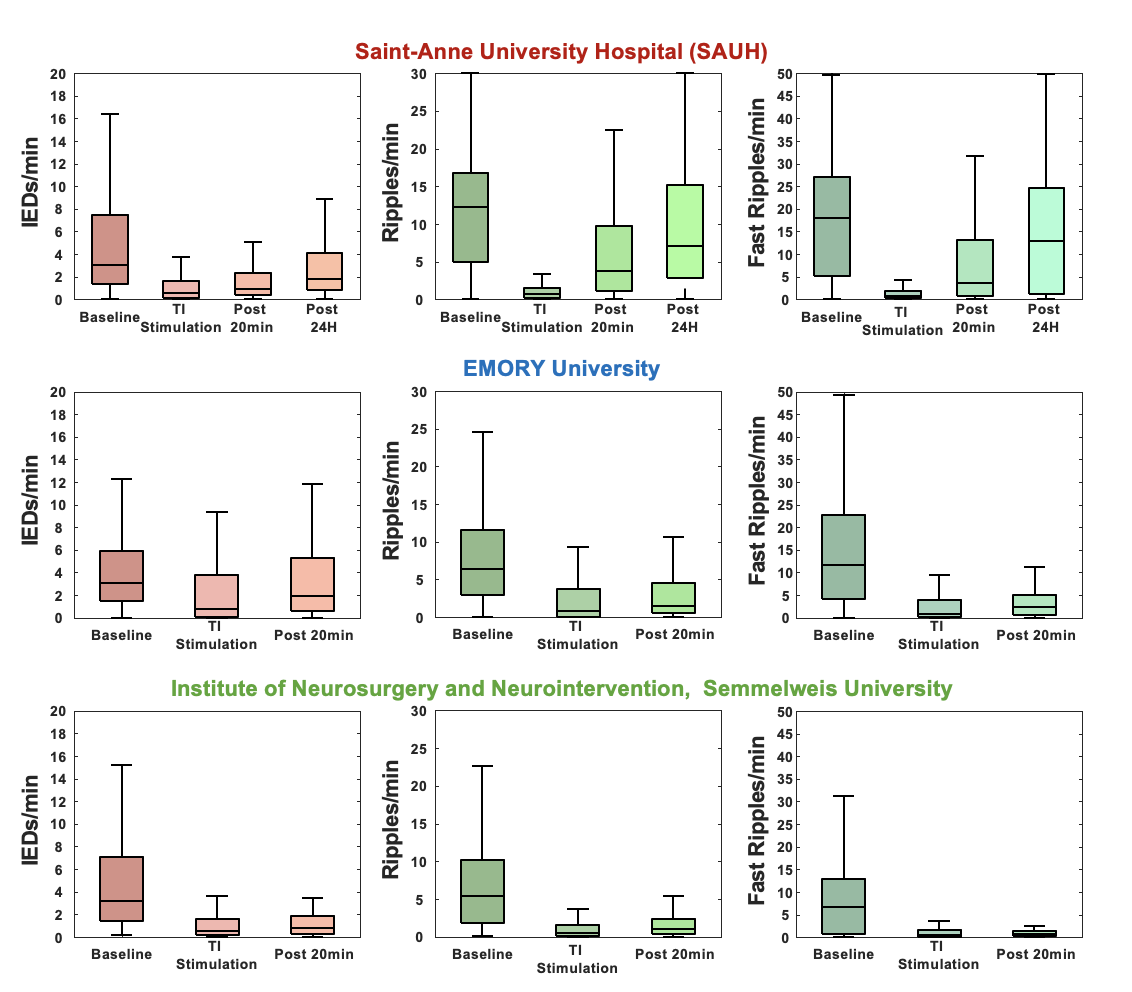
 The per-center cross-analysis of biomarkers show a similar trend within the 3 hospitals enrolled in the study. No center-specific difference (ANT ANOVA, rate ~ time x **center** x stimulation, p-value > 0.05 for center interactions) was observed for any of the biomarkers analyzed.


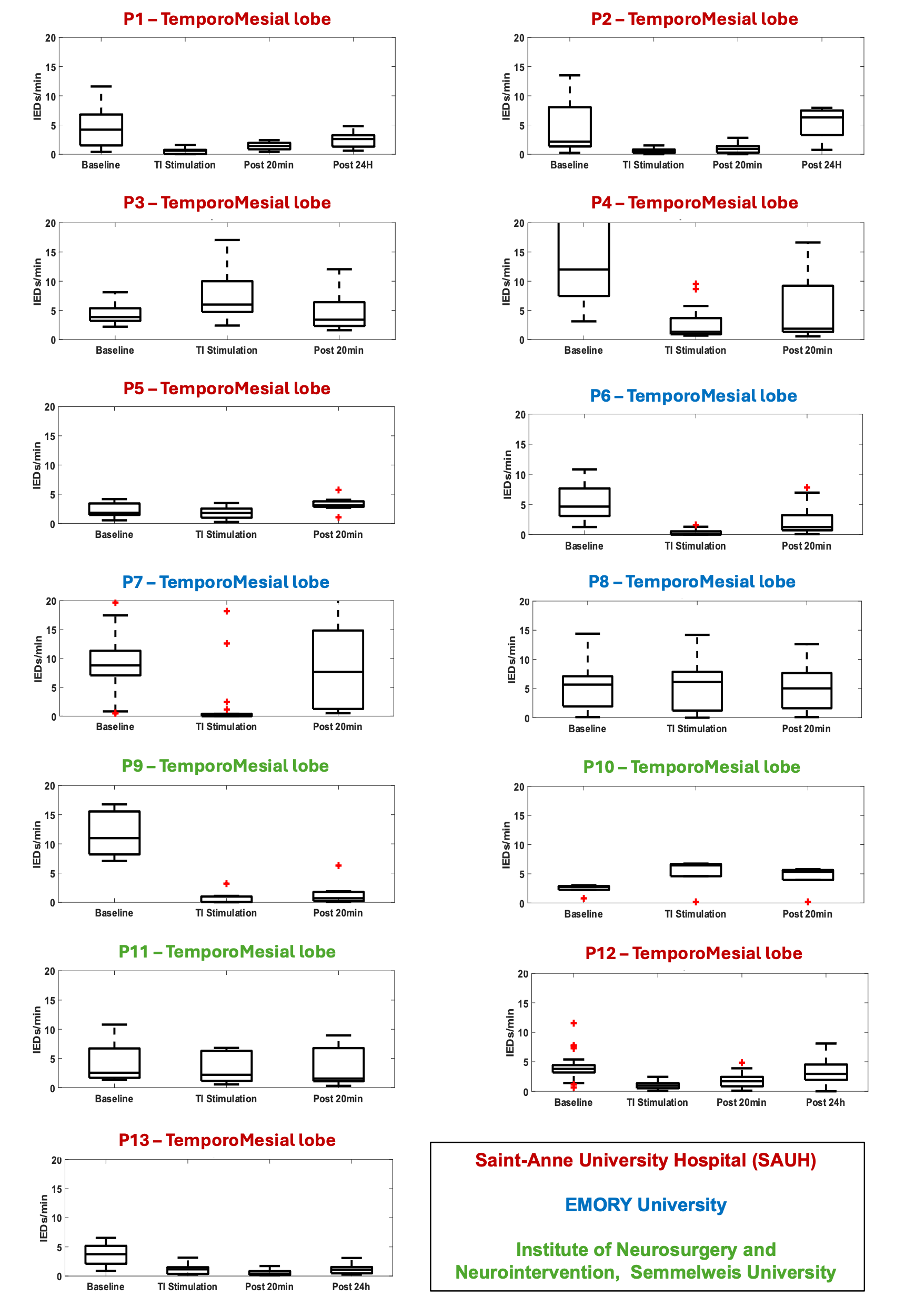


**Figure S2. Single patient interictal epileptiform discharge analysis.** The per-patient analysis of IEDs show a global trend within all patients but four enrolled in the study. TI stimulation decreases IEDs, and a carry-over effect is observed after the stimulation. This effect goes away within the first 24h following the stimulation.


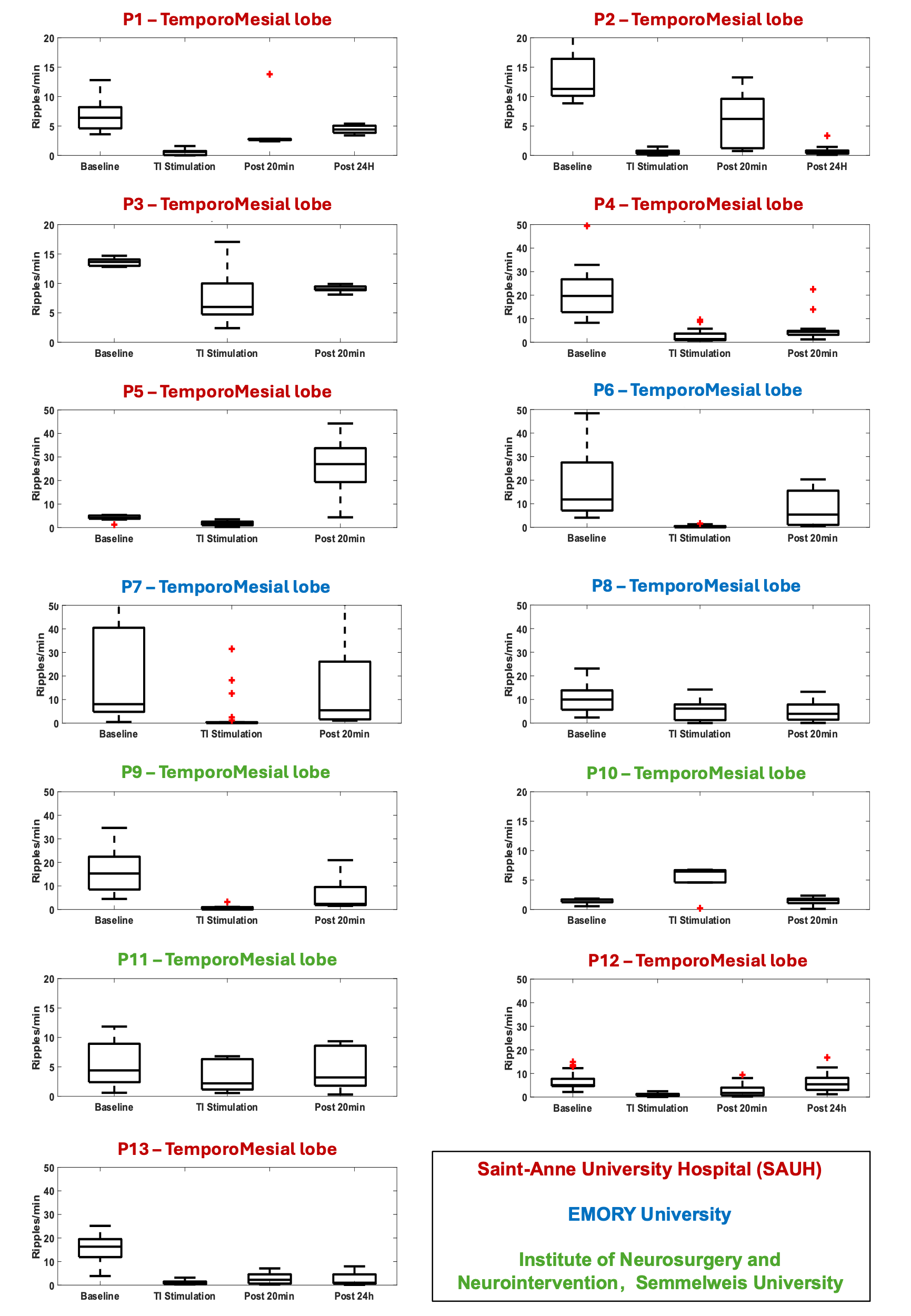


**Figure S3. Single patient ripples analysis.** The per-patient analysis of ripples shows a global trend within all patients but one enrolled in the study. TI stimulation decreases ripples, and a carry-over effect is observed after the stimulation. This effect goes away within the first 24h following the stimulation.


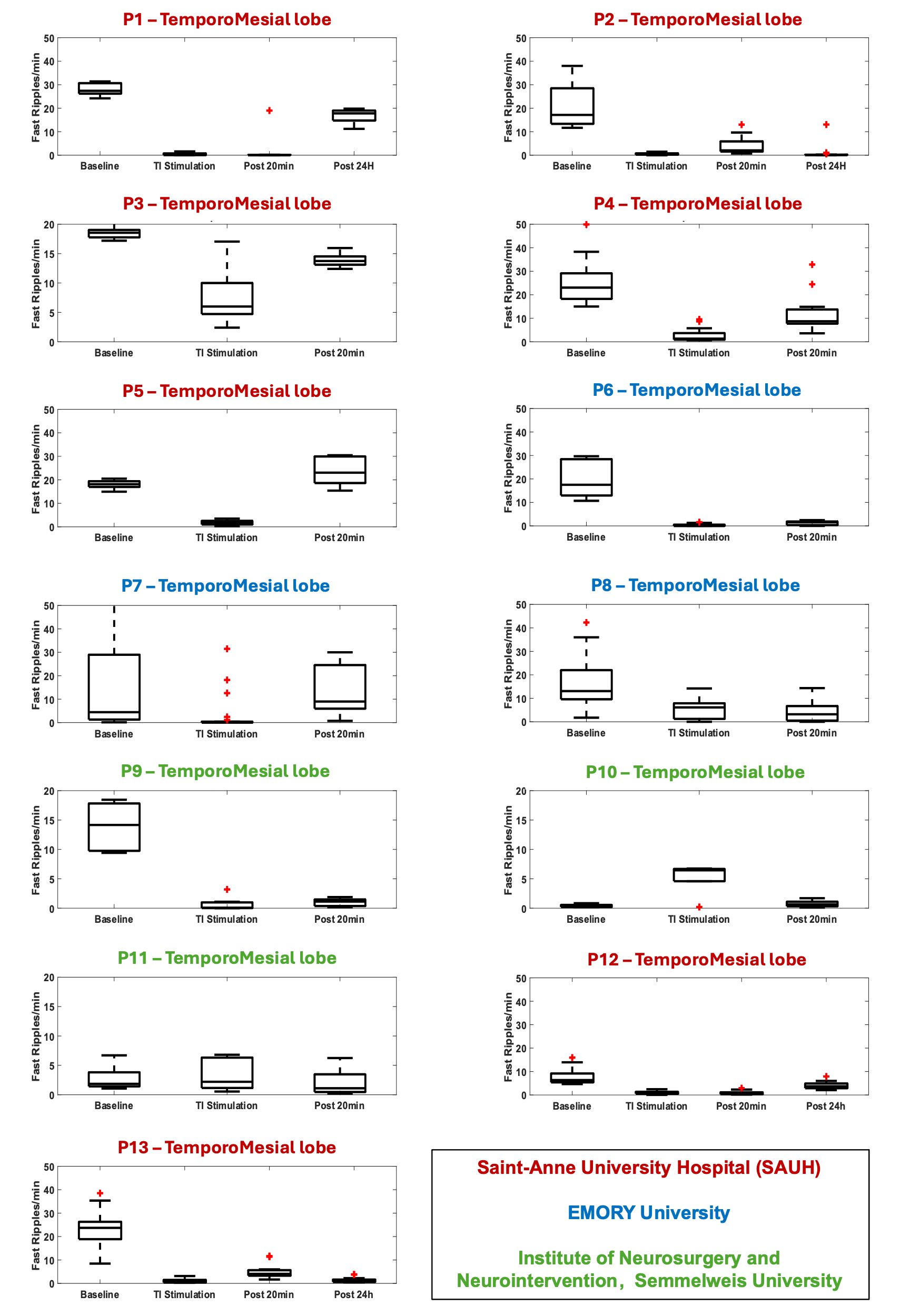


**Figure S4. Single patient fast ripples analysis.** The per-patient analysis of fast ripples shows a global trend within all patients but two enrolled in the study. TI stimulation decreases fast ripples, and a carry-over effect is observed after the stimulation. This effect goes away within the first 24h following the stimulation.

**
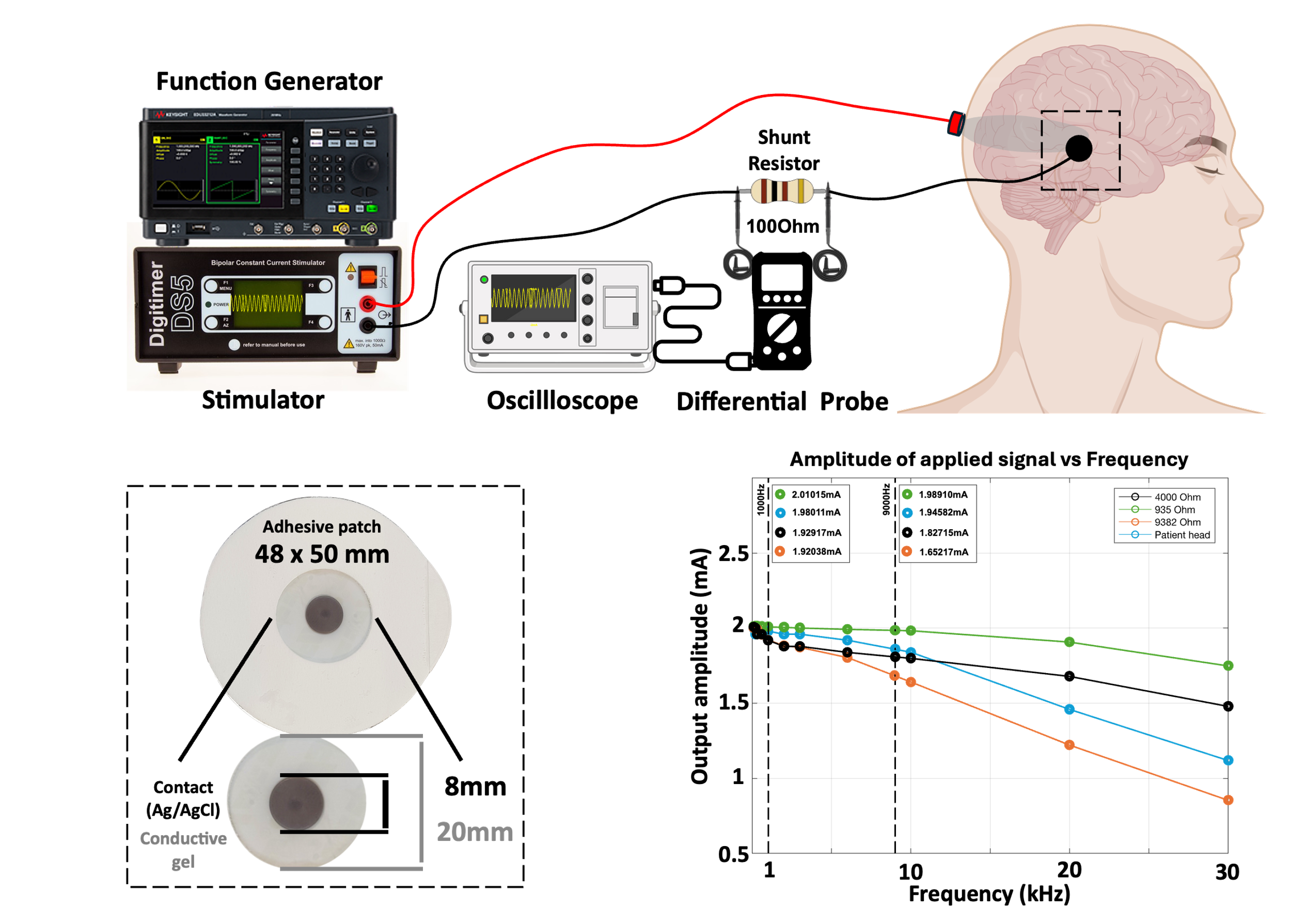
**

**Figure S5. Simulations of the TI stimulation for different carrier frequencies.** Measurement across a 100Ohm resistor of the output current as a function of the frequency applied via the Keysight and DS5. The relationship shows no significant applied current difference between lower carrier values (1000Hz) and higher carrier values (9000Hz) for a biological impedance (patient head – blue trace), effectively demonstrating the devices show no frequency roll-off effects from the spectrum of carrier values and impedances that were used in the study.


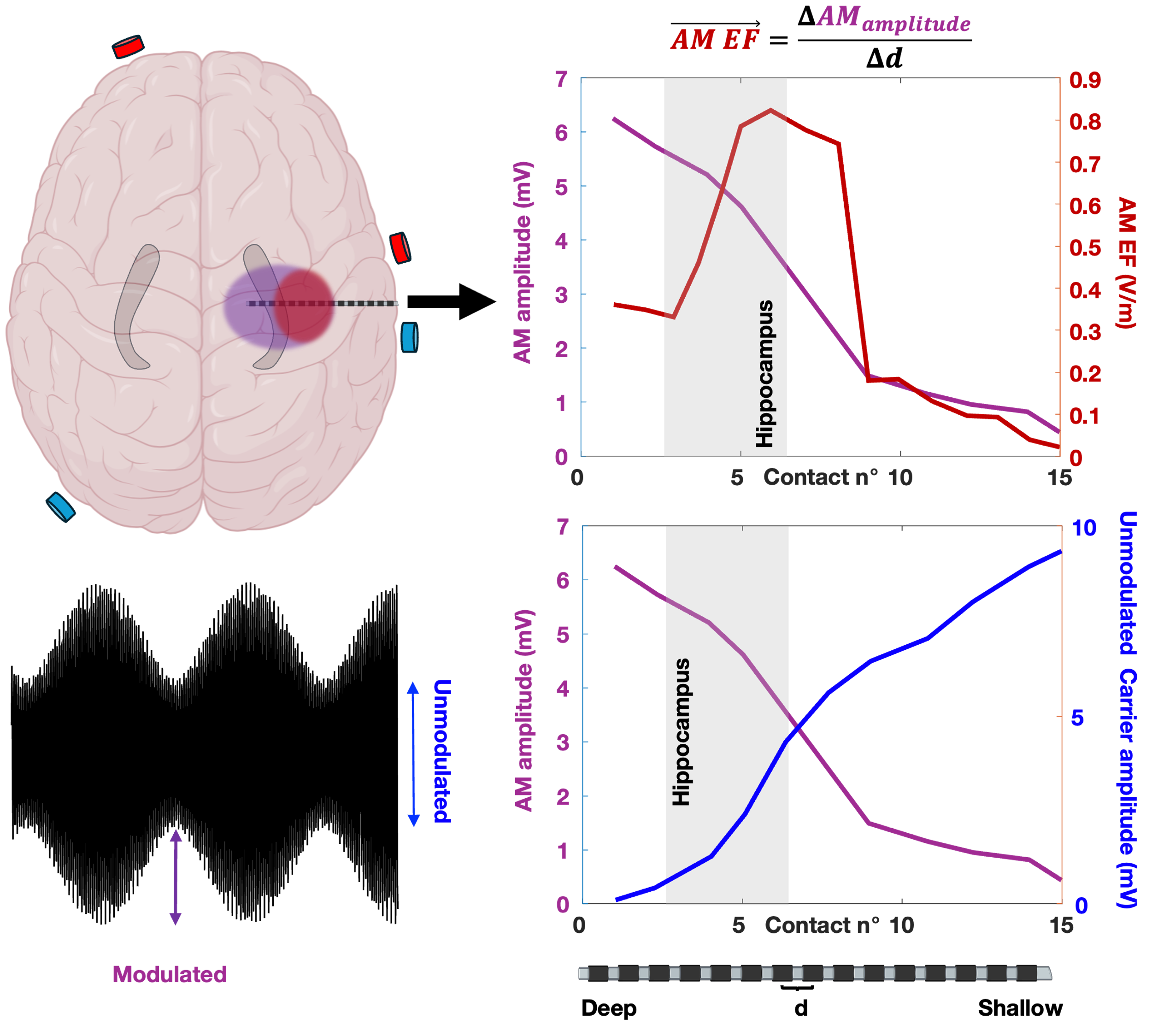


**Figure S6. Recorded AM amplitude and calculated AM EF along an sEEG electrode targeting the hippocampus.** The distribution of the maximum modulation amplitude and the maximum modulation electric field will differ. The experimentally measured maximum of the AM corresponds well to the maximum of the AM electric field (computed as the modulation magnitude of the difference of potential between two contacts) at the focus of the epilepsy in the hippocampus. (**top panels**) The source of the neuromodulation effect is assumed to be the maximum AM field exposure. To better investigate the distribution of waves in the brain, we compared AM distribution with Carrier distribution and showed a reverse trend where carrier frequency amplitude decreases, and AM amplitude increases with depth (**bottom panels**).

**
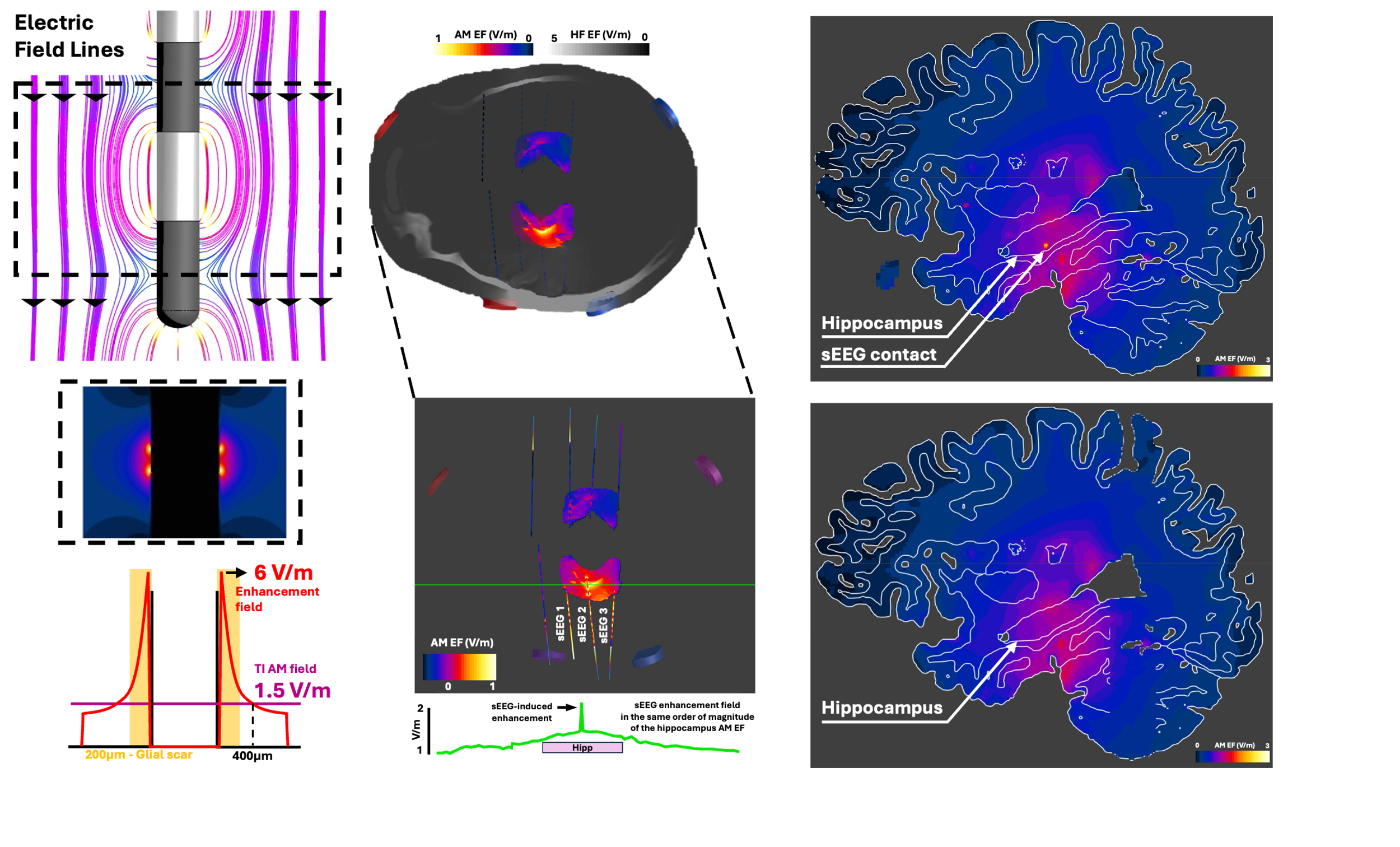
**

**Figure S7. Theoretical Field Enhancement around sEEG electrodes.** We characterize the electric field enhancement around the implanted SEEG electrodes during transcranial stimulation and determine the expected impact to be negligible. **(left panel - top)** Currents injected by transcranial stimulation can leverage low-impedance pathways through metallic SEEG contacts, and field lines bend near metallic surfaces such that tangential field components vanish. This not only results in local field enhancement^51^ near contact edges and bridging currents between neighboring contacts, but also in alignment of the two TI fields, resulting in an associated modulation magnitude increase. **(left panel – bottom)** The enhancement of the field was computed and found to be 2 to 7.2x the size of the incident TI modulation magnitude but decayed to the level of the surrounding AM field within 400 µm. Glial sheath formation around implanted intracranial electrodes is estimated to range from 150 to 300 µm. **(middle panels)** Visualization of the enhancement in a patient model – horizonal slice: the TI field near SEEG contacts in the hippocampus is depicted and plotted along the green trajectory. Several highly localized spots of AM enhancement are apparent. **(right panels)** Similar enhancement is evident in a sagittal cross-section. As can be seen in the middle and right panels, the entire hippocampus (left side) is covered by high AM exposure. The suppression of epileptic biomarkers at the brain network level, as observed in the current study, is unlikely to be caused by the highly localized subthreshold modulation near electrode contacts, considering that no significant enhancement is present outside the typical glial sheath thickness.


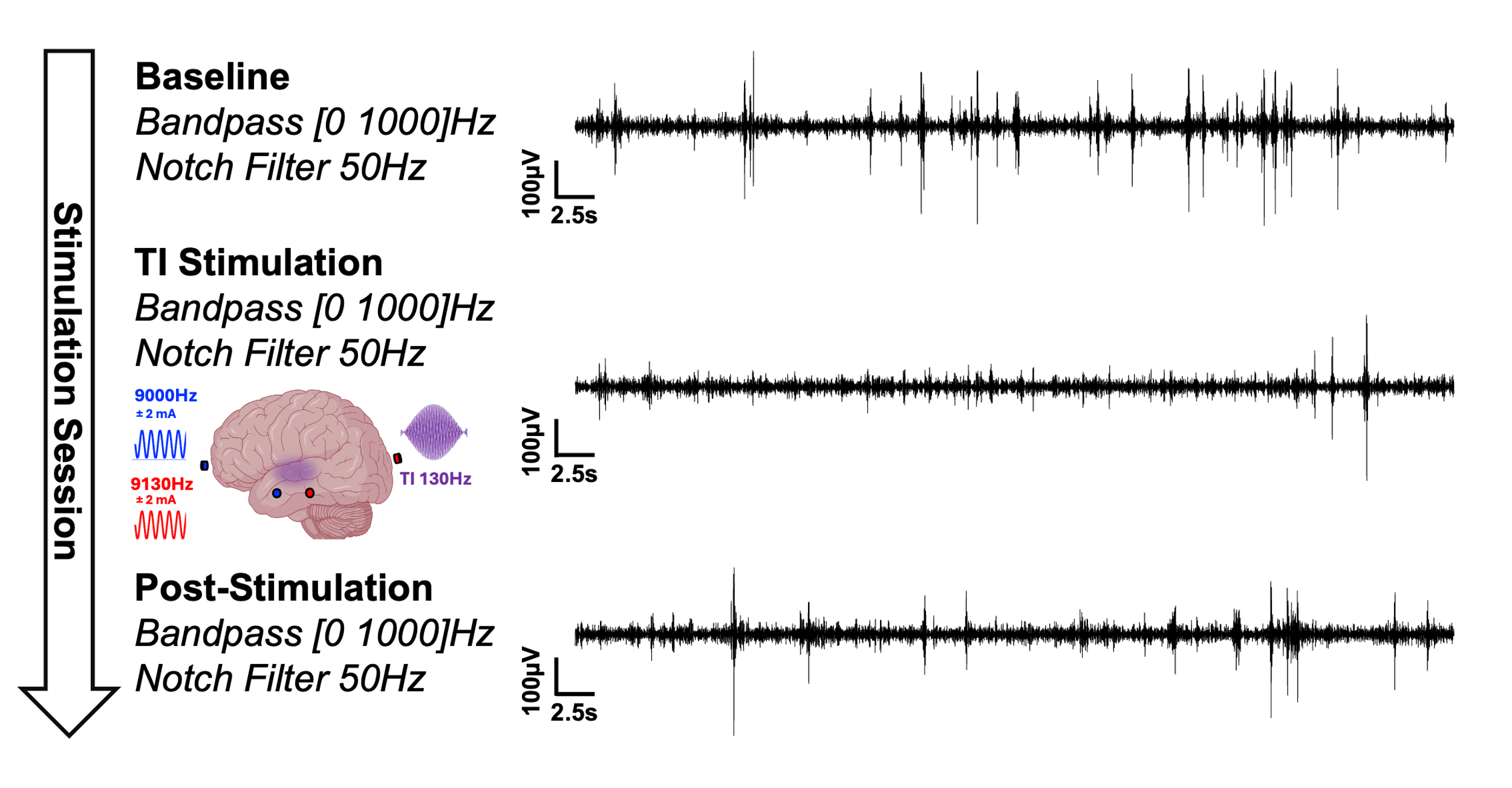
**Figure S8. Representative sEEG signals for Baseline, Stimulation and Post-Stimulation.** Brain activity was recorded during the whole protocol including Baseline recording where IEDs and HFOs were highly present, a TI stimulation recording – recording during the stimulation – with a significant decrease of epileptic biomarkers and a Post-Stimulation recording showing a carry-over effect for biomarkers suppression.

| **Patient** | **Age** | **Sex** | **Center** | **Head ∅** | **N° of electrodes** | **SOZ** | **Day PI** | **Target hippocampus** |
| --- | --- | --- | --- | --- | --- | --- | --- | --- |
| P1 | 35-39 | F | SAUH | 59 | 8 | Right temporal lobe | 6 | Right |
| P2 | 40-44 | M | SAUH | 61 | 13 | Left Insular lobe | 6 | Left |
| P3 | 35-39 | M | SAUH | 60 | 11 | Left temporal lobe | 6 | Left |
| P4 | 45-49 | F | SAUH | 60 | 8 | Bi-temporal lobes | 6 | Left |
| P5 | 40-44 | M | SAUH | 61 | 8 | Left temporal lobe | 6 | Left |
| P6 | 35-39 | M | EMORY | 54 | 10 | Bi temporal lobe | 7 | Left |
| P7 | 25-29 | M | EMORY | 60 | 13 | Left parietal lobe | 7 | Left |
| P8 | 35-39 | M | EMORY | 58 | 18 | Left temporal lobe | 7 | Left |
| P9 | 30-34 | F | INN-SU | 58 | 8 | Left temporal lobe | 8 | Left |
| P10 | 40-44 | M | INN-SU | 61 | 6 | Left temporal lobe | 7 | Left |
| P11 | 45-49 | F | INN-SU | 60 | 7 | Right temporal lobe | 6 | Right |
| P12 | 25-29 | M | SAUH | 61 | 12 | Right temporal lobe | 6 | Right |
| P13 | 30-34 | F | SAUH | 60 | 14 | Left temporal lobe | 6 | Left |

**Table 1. Participant characteristics.** Ages are displayed in ranges of 5 years to ensure anonymization of the patients. All patients enrolled in the present study showed a temporal lobe epilepsy without clear seizure onset zone delineated at the time of the enrollment. None of the demographic factors were considered cofounding for this study.
